# The structural efficiency of the sea sponge *Euplectella aspergillum* skeleton: bio-inspiration for 3D printed architectures

**DOI:** 10.1098/rsif.2018.0965

**Published:** 2019-05-08

**Authors:** K. Robson Brown, D. Bacheva, R. S. Trask

**Affiliations:** 1 CT Imaging Laboratory, University of Bristol, 43 Woodland Road, Bristol BS8 1UU, UK; 2 Department of Mechanical Engineering, University of Bristol, University Walk, Bristol BS8 1TR, UK; 3 Department of Aerospace Engineering, Advanced Composites Centre for Innovation and Science, University of Bristol, University Walk, Bristol BS8 1TR, UK; 4 Hieta Technologies, Bristol and Bath Science Park, Bristol BS16 7FR, UK

**Keywords:** structural efficiency, lattice structures, X-ray micro-computed tomography, additive layer manufacturing, composite materials

## Abstract

In Nature, despite the diversity of materials, patterns and structural designs, the majority of biomineralized systems share a common feature: the incorporation of multiple sets of discrete elements across different length scales. This paper is the first to assess whether the design features observed in the hexactinellid sea sponge *Euplectella aspergillum* can be transferred and implemented for the development of new structurally efficient engineering architectures manufactured by three-dimensional (3D) additive manufacturing (AM). We present an investigation into the design and survival strategies found in the biological system and evaluate their translation into a scaled engineering analogue assessed experimentally and through finite-element (FE) simulations. Discrete sections of the skeletal lattice were evaluated and tested in an *in situ* compression fixture using micro-computed tomography (μCT). This methodology permitted the characterization of the hierarchical organization of the siliceous skeleton; a multi-layered arrangement with a fusion between struts to improve the local energy-absorbing capabilities. It was observed that the irregular overlapping architecture of spicule–nodal point sub-structure offers unique improvements in the global strength and stiffness of the structure. The 3D data arising from the μCT of the skeleton were used to create accurate FE models and replication through 3D AM. The printed struts in the engineering analogue were homogeneous, comprising bonded ceramic granular particles (10–100 µm) with an outer epoxy infused shell. In these specimens, the compressive response of the sample was expected to be dynamic and catastrophic, but while the specimens showed a similar initiation and propagation failure pattern to *E. aspergillum*, the macroscopic deformation behaviour was altered from the expected predominantly brittle behaviour to a more damage tolerant quasi-brittle failure mode. In addition, the FE simulation of the printed construct predicted the same global failure response (initiation location and propagation directionality) as observed in *E. aspergillum*. The ability to mimic directly the complex material construction and design features in *E. aspergillum* is currently beyond the latest advances in AM. However, while acknowledging the material-dominated limitations, the results presented here highlight the considerable potential of direct mimicry of biomineralized lattice architectures as future light-weight damage tolerant composite structures.

## 1. Hypothesis

Investigations into different biological systems have offered the physical sciences community a plethora of new ideas in the pursuit of more mechanical and energy efficient architectures to develop the ‘products of the future’. Beyond correctly characterizing the biological system (material, composition, architecture and functional role), the key challenge is the extrapolation of this new insight to engineering length scales, using current tools and existing manufacturing schemes for the targeted application in physical products for human endeavour. The study presented here explores this challenge; namely, the physical and mechanical characterization of a biological system (i.e. the skeleton of hexactinelid sponge *Euplectella aspergillum*), with the sole purpose of extrapolating this understanding, using existing state-of-the-art simulation and manufacturing approaches, to determine whether the underlying biological ‘design’ principles could be applied within the engineering domain. The authors accept biomimicry of such a complex biological system is beyond current engineering practice, but a critical question remains; namely can engineering materials in conjunction with a ‘bioinspired design philosophy’ develop the products of the future?

## 2. Introduction

Advanced composite materials offer the designer considerable design freedom and unique functional performance, such as low weight, high specific strength and stiffness, while offering unique damage tolerance and crashworthiness methodologies. Additionally, with developments in fibrous and polymeric materials, as well as new additive manufacturing (AM) processes (rather than subtractive), advanced composites offer the potential for the integration of system-specific multifunctional capabilities, whereby multiple structural and non-structural functions will reside within the backbone of the structure [1,2].

It is the on-going quest for light-weight, multifunctional and high-performance structural materials which is leading academic and industrial researchers to consider and study biological composites [3] as inspiration for future design concepts. Rigid natural systems such as bone, teeth, nacre and silica sponge are different in structure and composition, yet they share common design principles. The latter include hierarchical structure from nano to macro levels, adaptation of form and structure to function, a unique inorganic–organic composition from simple low mechanically performing structural elements, compared to synthetic systems and the presence of numerous interfaces resulting in highly efficient toughening mechanisms [4]. Through this strategy, mineralized biological composites possess high strength, stiffness and fracture resistance, a combination which is not fully achieved with engineering materials [3,5].

**Figure 1.**
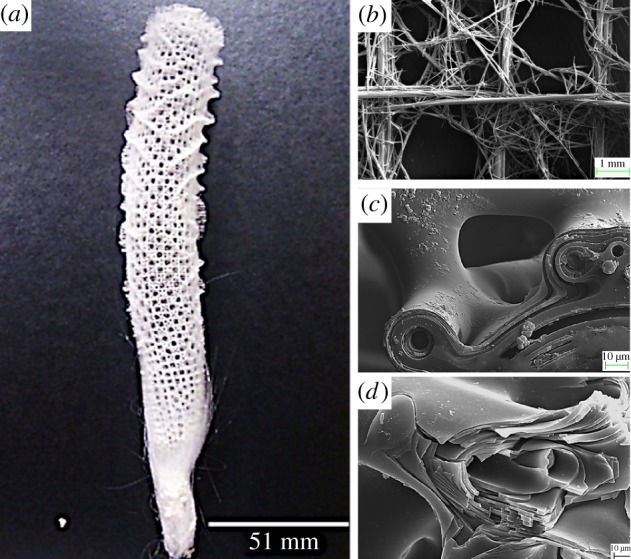
(*a*) *Euplectella aspergillum* cylindrical lattice skeleton. (*b*) Scanning electron microscopy (SEM) image showing the overlap of the stauractine spicules and the node, forming a triangulated area in the lattice interior and containing the axial spicule centre. (*c*) A magnified region of (*b*), showing the fusion connectivity between a principal cruciform spicule and a diactine spicule. (*d*) SEM image of a fractured spicule, indicating the creation of crack-stopping and crack-deviating mechanisms.

The hierarchical organization of the skeleton of hexactinelid sponge *E. aspergillum* (figure 1), a silica-based structure, is an excellent example of this design strategy for enhancing the performance of an inherently brittle material. The strength of amorphous silica is determined by the existence of surface flaws, and if its size goes beyond a few micrometres, there is a dramatic loss of strength. To overcome this limitation, and to build structures which are larger than a few micrometres, further hierarchical levels are required to ‘modify’ silica into an adaptive three-dimensional (3D) cylindrical skeletal lattice [6,7]. A full description of the structural hierarchy of *E. aspergillum* is beyond the scope of this communication, and the reader is referred to the detailed study by Weaver *et al*. [8]. However, in summary, and as figure 1 indicates, *E. aspergillum* is formed of two independent interwoven square lattices, constructed by laminated non-planar cruciform spicules formed of consolidated silica nanoparticles. It is the complex interactions across all these structural levels which yield the superior mechanical performance of the skeleton [8,9], and the motivation for this study.

In hexactinellid sponges, the structural loads exerted onto the spicular skeleton arise primarily through the surrounding ocean currents and the need to support the soft tissue. In this study, the mechanical response of the lattice skeleton to a compressive load is investigated, with the assumption that the latter acts as a static support. For this purpose, portions of the lattice of one sponge skeleton were extracted and compression tested *in situ* using X-ray micro-computed tomography (μ-CT). The combination of μ-CT and *in situ* mechanical loading is particularly useful for understanding the relationship between the morphology and mechanical behaviour of the biological system. In addition, the 3D imaging data obtained from μ-CT can be used for the creation of highly accurate meshes for finite-element (FE) modelling and AM/3D printing. In our study, the aim was to employ this methodology to determine if the damage mechanisms translate from the biological system to its engineering counterpart, noting the key limitation of using additive layer manufacturing and current 3D printing technology, namely the elimination of internal micro-architecture.

## 3. Material and methods

### 3.1. Specimen extraction

**Figure 2.**
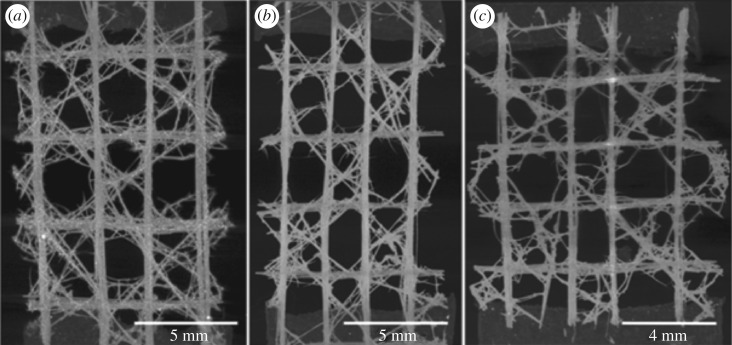
μ-CT maximum intensity projection images of the three different biological samples in *xz*-plane. (*a*) Closed Cell 1, (*b*) Closed Cell 2 and (*c*) Closed Cell 3.

Three 3 × 3 unit cell samples were extracted from the lower portions of three *E. aspergillum* specimens, using a scalpel. The samples were washed in distilled water and cleaned for 30 s in an ultrasonic cleaner, then dried at room temperature for 24 h (figure 2). Figure 2 illustrates the three different specimens with the caption detailing the surface volume values of the individual samples.

### 3.2. *In situ* loading and micro-computed tomography of the sponge skeleton

To secure the upright position of the sample and to ensure flat and parallel surfaces for compression testing, the upper and lower ends of the samples were cast in epoxy resin (EPON 828) with diethylenetriamine (DETA) as curing agent using a silicon mould. The *in situ* tests were conducted in the specimen chamber of a Bruker SkyScan Material Testing Stage (MTS-50 N), with the lower end fixed to the bottom holder and the upper one free (see the electronic supplementary material, figure S1). The testing stage was positioned inside the μ-CT scanner (Bruker SkyScan 1172, Belgium) with the sample loaded in compression at 2 µm s^−1^, in several incremental steps. All the scans were performed at a resolution of 5 µm per pixel prior to loading and after each loading cycle. The reconstruction of the grey scale images into cross-sectional image slices was performed with NRecon v.1.6.8 (Bruker SkyScan, Belgium) reconstruction software. The image stacks of each scan were segmented, smoothed with a convolution filter and visualized with VGStudio Max 2.1.

### 3.3. 3D printing the engineering analogue

**Figure 3.**
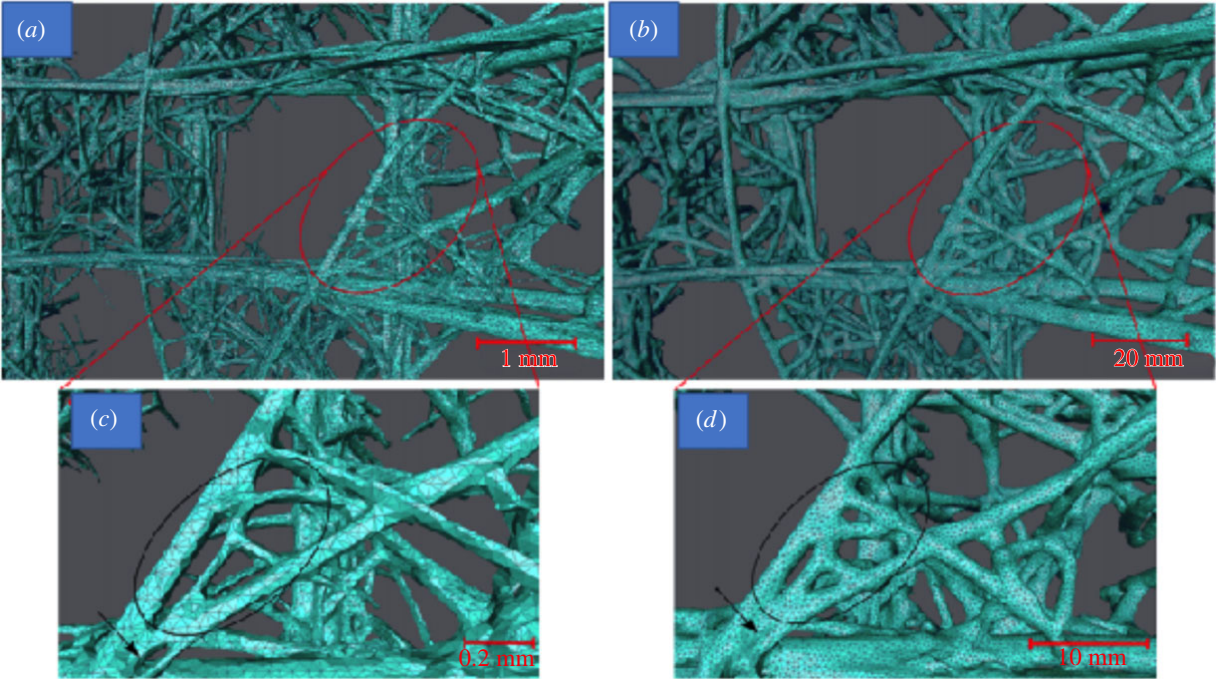
Images showing the surface mesh generation of the stereolithography model. (*a*) The simplified polygonal surface; (*b*) the final mesh of the stereolithography model after offset, manual editing and remeshing. Red rings indicate zoomed areas of (*c*) the simplified and (*d*) the final mesh. Black rings show the same region of the mesh before and after mesh editing. The increase in strut thickness and consequent decrease in the distance between the struts can be clearly seen. (Online version in colour.)

In order to create an engineering analogue, the raw data from the biological system were used to generate a stereolithography model (.stl) in VGStudio Max. The .stl file generated from the biological system was too computationally expensive for further processing and, as a result, the polygonal mesh was simplified with the ‘Simplification Editor’ function in Avizo Standard v. 7.1 (FEI Visualization Sciences Group, Bordeaux, France). Although effective in reducing the computational expense, the mesh simplification resulted in smoothing some of the features of the object (e.g. the number of faces within the closed cell model reduced from 65 984 402 to 1 000 224) leading to the introduction of irregular surfaces consisting of poorly shaped polygons and intersections (figure 3*a*,*c*). While significantly improving run time, these limitations necessitated some operator intervention to restore some elements of the lattice topology.

In order to comply with the 3D printing (3DP) requirements, the mesh was scaled 20 times the original model and offset using the ‘Uniform Mesh Resampling’ in MeshLab v1.3.2. A new triangulated surface was created with a precision and offset of 0.63 and 0.20 in absolute values, respectively. This allowed a mesh expansion without compromising the overall sponge lattice topology. The resampled mesh was imported into GeoMagic Studio13. The wall thickness of the struts was manually increased using the ‘Offset Polygons’ function preserving the features of the sponge lattice. The final preparations for 3DP included smoothing and remeshing the surface to eliminate the mesh distortions, which were present in the model due to the localized polygon offset (figure 3*b*,*d*). The edited mesh was then imported into ZPrint 7.10 software for 3DP using a ZCorp Spectrum Z510 printer and zp150 high-performance powder (calcium sulfate hemihydrate particles (ranging in size from 10 to 100 µm in diameter and variable length) with a polyvinylpyrrolidone adhesive binder). This powder is characterized with a higher green strength compared with other generation ZCorp powders, which makes it suitable for handling of complex printed structures which consist of numerous thin struts.

**Figure 4.**
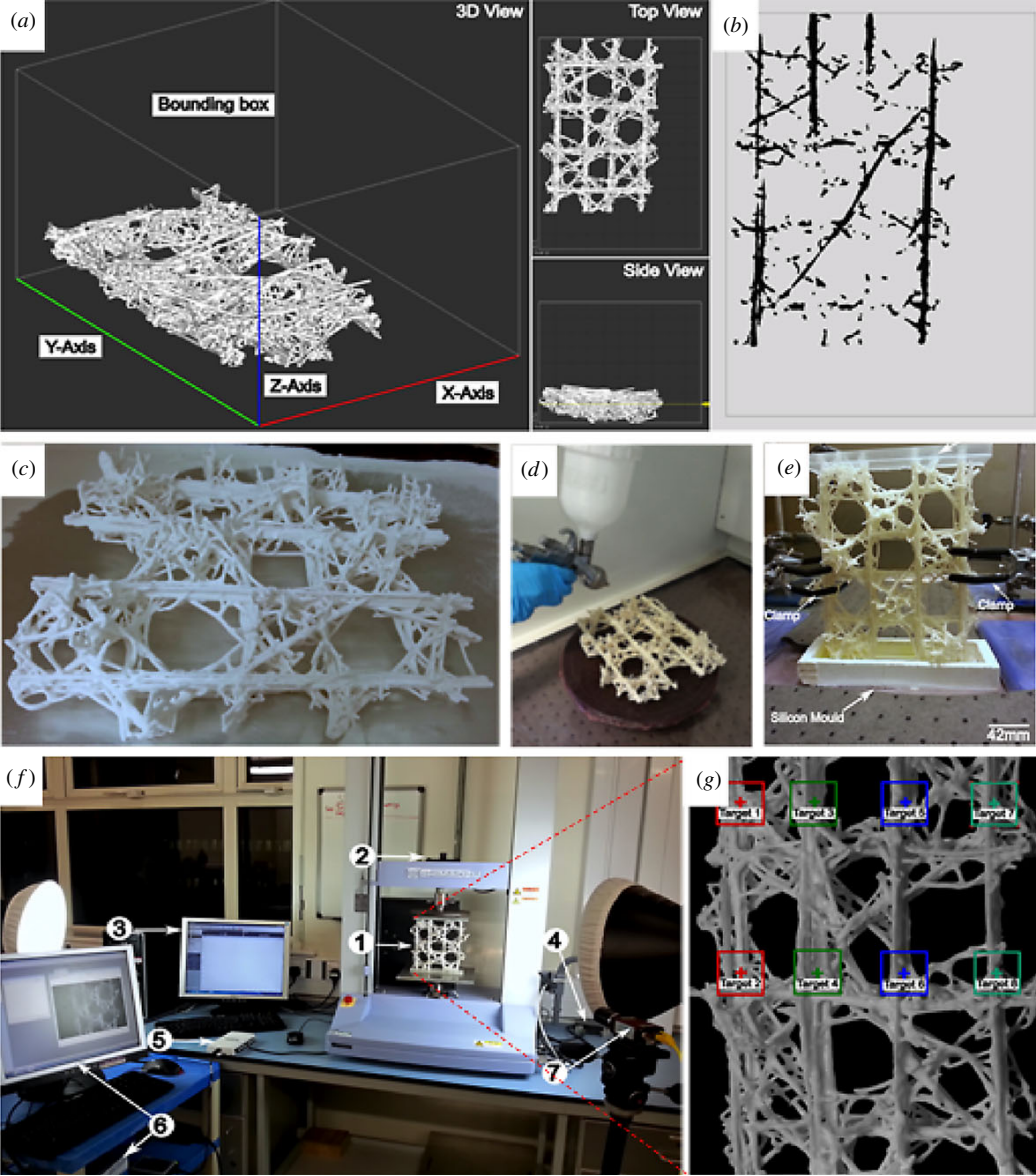
Design, build and testing of 3D printed sample. (*a*) ZPrint main window showing the orientation of the part in the build bed in 3D view. (*b*) Top view showing the contours of an individual slice, the location of which is indicated by the yellow slider in the side view of (*a*). (*c*) The 3DP specimen directly after de-powdering process, i.e. the removal of the loose powder between the lattice cell and elements. (*d*) Manual spraying process of 3DP samples. (*e*) Epoxy end potting of specimens in preparation for testing. (*f*) Experimental set-up and instrumentation: (1) sample; (2) load cell; (3) computer with Trapezium X testing operation software; (4) analogue output module, which outputs the load as an analogue signal from the test machine to the NI data acquisition module (5); (6) Imetrum Video Gauge system and software; (7) high-resolution digital camera connected to the video gauge system. (*g*) Paired target locations for Imetrum video extensometer to record relative displacement during loading. (Online version in colour.)

A further consideration when generating 3D printed architectures concerns the print layer orientation and the influence this will exert on the mechanical properties of the final part [10,11]. The closed cell models were oriented with the longest axis along the *y*-direction of the build bed, which is the printer head's direction of travel (figure 4*a*). The orientation was chosen according to the recommendations for the highest ultimate green strength for 3D printed parts, the direction of the applied uniaxial compression load and the longest dimension of the models (figure 4*a*,*b*). In the 3DP process, the print orientation governs the mechanical performance of the part; where the strongest performance is oriented along the *y*- and *x*-axes with the least along the *z*-axis. After printing, the sample was depowdered to remove the loose material, deposited around it during printing (figure 4*c*). To improve the comprehensive strength of the sample, the porous architecture was subsequently infiltrated and stabilized with ZMax epoxy resin using a high-volume, low-pressure spray gun (figure 4*d*). Three coats were applied in the direction of the vertical beams and two in the direction of the horizontal beams on each side of the sample to ensure efficient resin penetration through the thickness of the struts; followed by a cure for 2 h at 71°C. In a similar manner to the biological samples, the ends were cast in epoxy resin (EPON 828) with DETA as curing agent using a silicon mould (figure 4*e*). The sample was subjected to a compression loading on a Shimadzu Autograph AGS-X Series testing machine with a loading rate of 1 mm min^−1^, connected to an Imetrum video extensometer (figure 4*f*,*g*) with a resolution of 2.8 µm ± 5% [12]. The latter is an efficient non-contact method for strain/displacement measurement, operating by precisely tracking the position of specific features or predefined targets on the specimen's surface in video images. The video records the movement of the target during test, while the software extracts the strain or displacement values by measuring the target movement.

### 3.4. Finite-element analysis

The FE model was based on surface topology of the 3 × 3 Closed Cell 1 (figure 2*a*) for the manufacture of the physical samples. The FE simulation was performed with explicit LS-DYNA code, version LS971vR6.0.0 (Livermore Software Technology Corporation, Livermore, USA). LS-DYNA provides a wide range of material models to capture different types of material behaviour, offering highly nonlinear, robust and computationally efficient contact algorithms. Furthermore, within LS-DYNA several constitutive models are employed to capture the fundamental behaviour of multiphase materials, for example, post-peak softening, shear dilation, strain rate and confinement effects. In this study, the parts were assembled in Oasys PRIMER 10.2 64bit (Ove Arup SYStems) pre-processing software. The geometry of the loading plate was created in MSC Patran 2012 64-bit (MSC Software Corporation, Newport Beach, USA) [13] with dimensions 300 × 300 × 25 mm and discretized with 21 609 nodes and 18 432 eight-noded solid brick elements. The part was exported as LS-Dyna file (.KEY file) and modelled as a rigid body. The tetrahedral meshes with the Closed Cell configuration were imported as .IDEAS file into Oasys PRIMER and exported as separate KEY files. The simulation required an efficient and accurate material model to capture: (1) the transition from linear to nonlinear elastic regimes, (2) the subsequent nonlinear strain hardening (yielding), and (3) the post-peak strain softening. To meet these requirements, the Karagozian & Case Concrete (KCC) Model—Release III was selected to simulate the behaviour of the 3 × 3 Closed Cell model subjected to uniaxial compression loading. The KCC model is regarded as a three-invariant mode [14], which falls under the generalized isotropic plasticity theory, and comprises elastic and plastic updates, strength surface formulations, rate and scale effects and damage accumulation parameters [15]. It differentiates the deviatoric and volumetric responses and also requires an equation of state. KCC uses a three-surface plasticity formulation which incorporates a material damage evaluation algorithm to compute, at each time step of the simulation, a new failure surface. To calculate the failure response, the KCC model requires the experimentally defined unconfined uniaxial compression strength and the material density. The compression strength was obtained from the authors' uniaxial compression testing of 3D printed cylinders. The obtained values for the uniaxial compression strength and the density are 39.7 ± 4.5 MPa and 1.60 ± 0.02 g mm^−3^, respectively. In LS-DYNA, these experimentally derived data are directly included as *MAT CONCRETE DAMAGE REL3 [15] with the default parameters being obtained from an extensive range of experimental tests, including unconfined uniaxial compression and tension tests, triaxial compression tests and a number of strain path tests [16,17]. The author defined data in conjunction with the default parameters allow the KCC model, with a mesh element size of 1.5 mm, to accurately capture post-peak softening, shear dilation, confinement and strain rate effects of the closed cell system. The reader is referred to the electronic supplementary material for full breakdown of the derived and default parameters used within the FE simulation.

## 4. Results

### 4.1. The failure behaviour of the *Euplectella aspergillum* 3 × 3 unit cell

**Figure 5.**
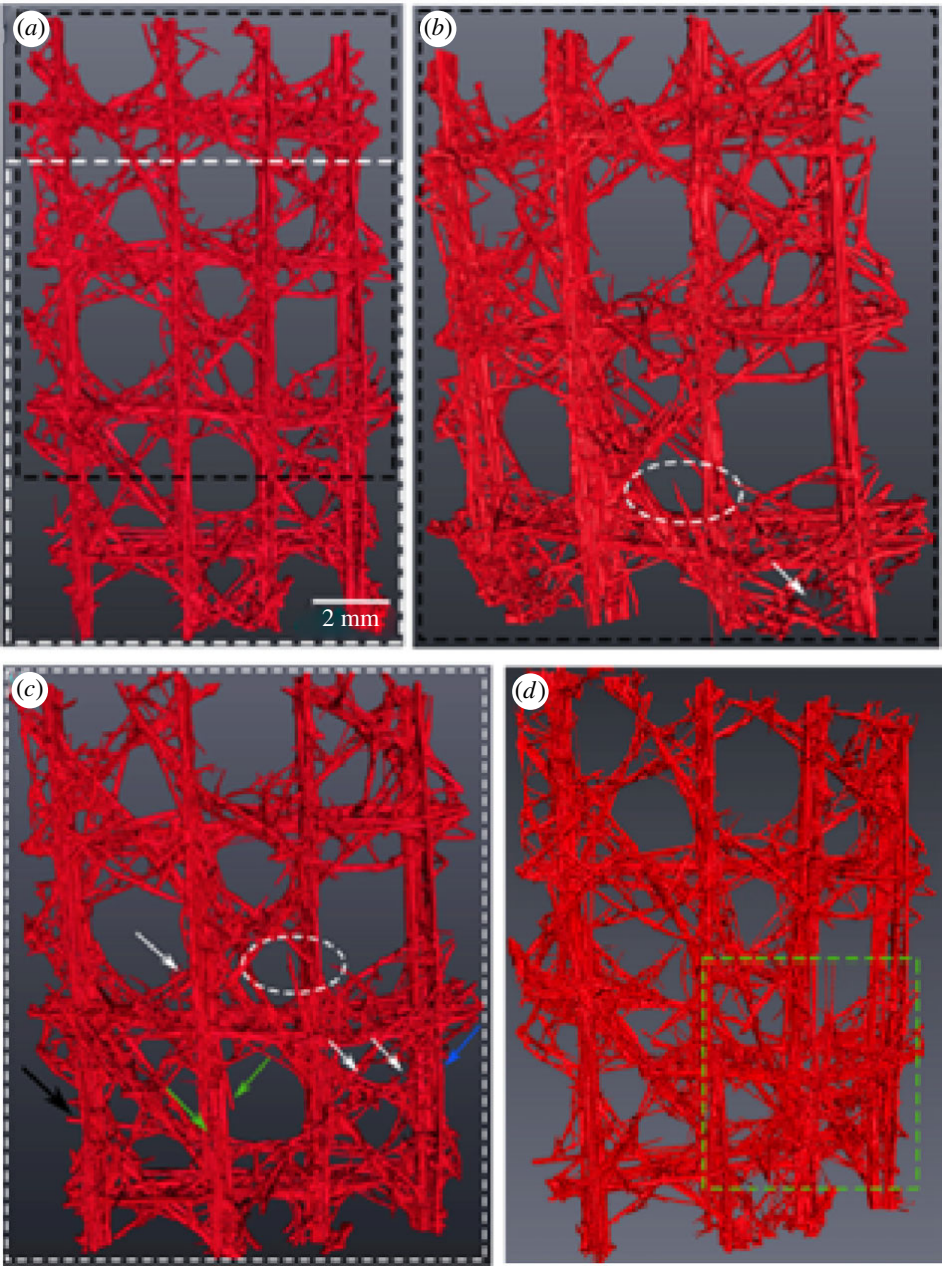
3D surface renderings of *in situ* compression scans of Closed Cell 1 sample, at the four different loading points. (*a*) Scan of whole specimen prior to loading. (*b*) First scan at 50% maximum load showing initial damage formation. White dashed circle illustrates fracture and subsequent delamination of the surface reinforcing spicules from the main beams. White arrow points to fractured diagonal spicules. (*c*) Second scan at peak load. Arrows indicate fracture of the reinforcing spicules. (*d*) Third scan showing densification of the lattice cells and delamination of the horizontal beams (green box). (Online version in colour.)

The average failure for all three 3 × 3 specimens was determined as 14.3 N with a standard deviation of 2.8 N. The average extension at the onset of catastrophic failure was recorded as 1.1 mm with a standard deviation of 0.2 mm. Figure 5 shows the reconstructed images at four different loading points illustrating the failure behaviour of Closed Cell 1 specimen during the *in situ* compression testing. The description of the failure response, given below, is typical of all the specimens.

**Figure 6.**
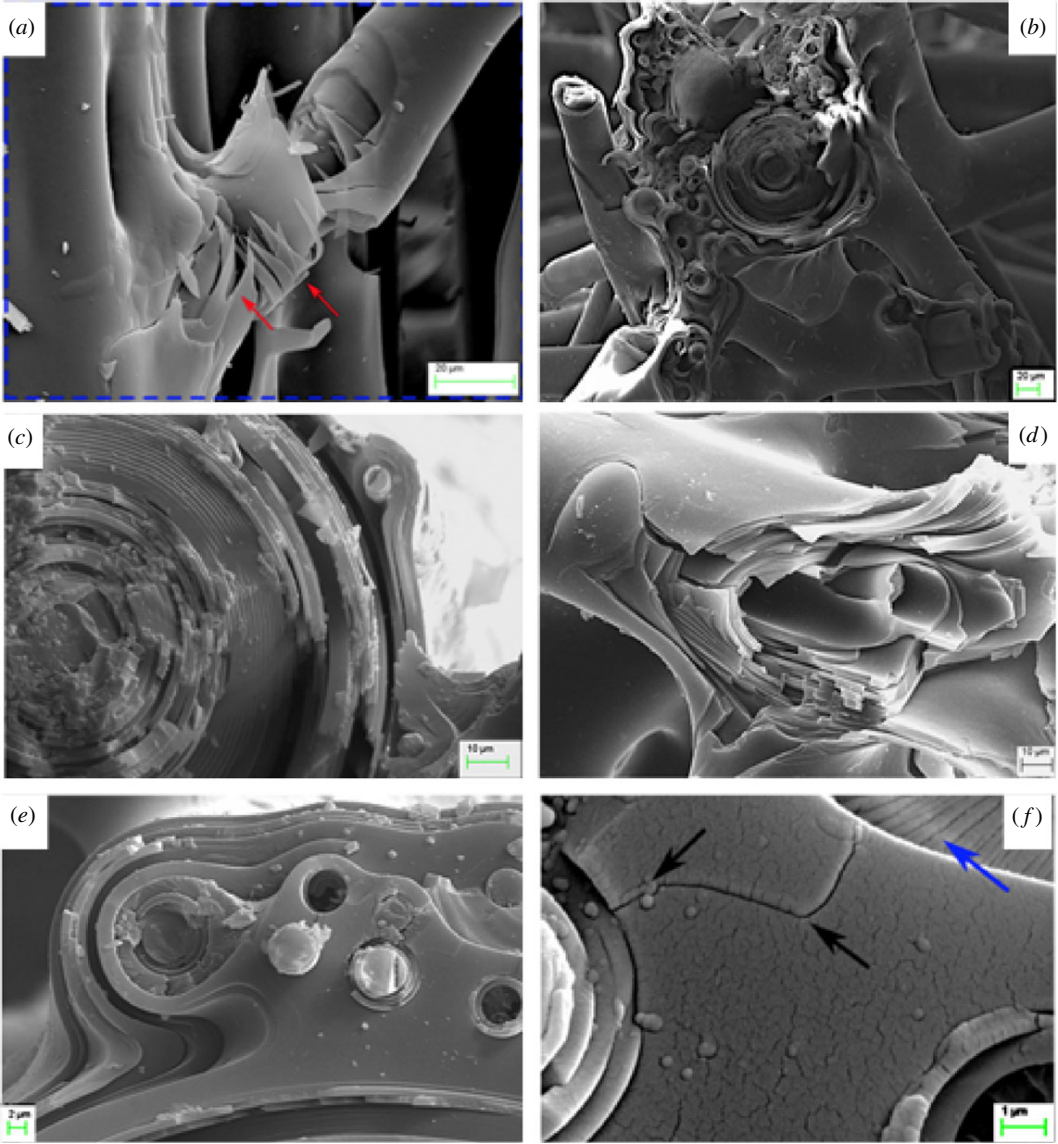
Observed hierarchical failure modes in *E. aspergillum* 3 × 3 unit cell through scanning electron microscopy. (*a*) Fracture of the lattice framework through vertical splitting, fracture in the diagonal arms and supporting silica matrix (red arrows), leading to (*b*) complex fracture paths in the main load-bearing vertical beams. (*c*,*d*) Laminated spicules promote a complex layered/radial fracture path which enhances the damage tolerance of the entire skeleton. (*e*) Efficient crack-stopping mechanism at nodal connection points. (*f*) Cracks diverged by small spicules embedded in the silica cement (black arrows) and fracture lances formation (blue arrow). (Online version in colour.)

The lattice initially exhibits an initial stiff linear response characterized with no visible damage to the main struts. The first fracture occurs in the right reinforcing vertical strut and in a diagonal beam near the bottom section (figure 5*b*, white arrows). Although, the main beams remain intact, the deformation of the upper parts of the outer vertical struts was readily observed during this phase of the test. The yield point (the onset of strut buckling or crushing at the peak load, figure 5*c*) is followed by a load drop, post-yield softening and load plateau. The observed progressive nature of the fracture corresponds to the failure of the lattice components, crack deviation and arrest in the silica cement and strut–beam connection point failure (see figure 6*a–f* indicating the different failure modes). As the compressive load progresses towards final failure (lattice densification), the shear fracture of the main vertical beams was clearly observed in each experimental sample. This led to the failure of the diagonal reinforcing struts, breaking away from the main vertical beams (figure 6*a*), which the authors deemed responsible for the loss of stability and subsequent fracture of the main vertical beams (figure 6*b*–*d*) and the delamination of the horizontal beams. In all specimens, the brittle crushing of the longitudinal beams takes place near the nodes between the mid and lower cell rows and the mid and upper rows. It is noticeable that the fracture develops in shear pattern, predominantly in the cells with lower connectivity, i.e. the open cells.

According to Maxwell's rule [18], a square lattice with rigid nodes is characterized with a failure in bending of the constituent struts, hence the lattice behaves as a bending-dominated structure. The introduction of diagonal elements increases the nodal connectivity, resulting in a lattice with stretching-dominated failure mode [10,19,20]. The role of the diagonal elements, whether under tension or compression loading, is to redistribute the loads. In hexactinellid sponges with main quadrate skeletons, the introduction of long diagonal elements that intersect the nodes appears to be in conflict with sponge hydrodynamic efficiency. Nonetheless, the diagonal beams still re-channel the loads and are under tension or compression depending on the load direction. They also act to reduce the stresses away from the nodal intersections by delocalizing the stress to neighbouring beams. This arrangement is highly beneficial for rigid frameworks since most of the stresses are concentrated at the nodes and in the middle of the beams. In addition, this configuration decreases the effective length of the longitudinal beams, thus preventing buckling of the latter under compressive loading [20]. Force–displacement plots are available as open data at doi.org/10.5061/dryad.ns7n1/1. Experimentally, it is clear that this strategy is adopted by *E. aspergillum* sponges resulting in a damage tolerant and rigid supportive skeleton.

### 4.2. The failure behaviour of the 3D printed engineering analogue

**Figure 7.**
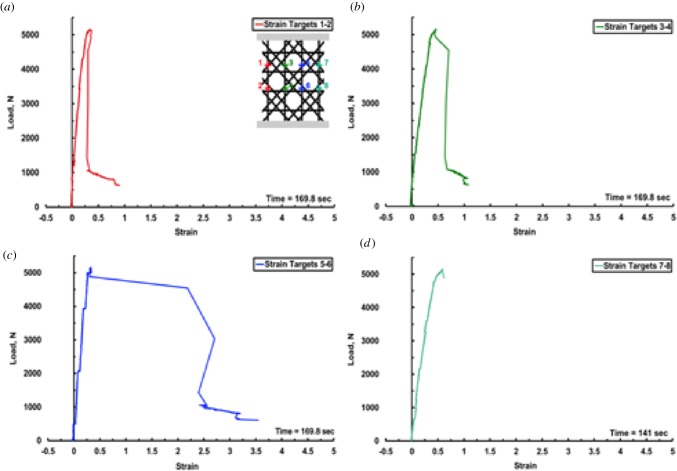
Load versus vertical strain measured on 3D printed engineering analogue. (*a*) Load–strain response between Target 1 and Target 2. (*b*) Load–strain response between Targets 3 and 4. (*c*) Load–strain response between Targets 5 and 6. (*d*) Load–strain response between Targets 7 and 8. The displayed time in each panel is the time at which the strain targets were lost. (Online version in colour.)

**Figure 8.**
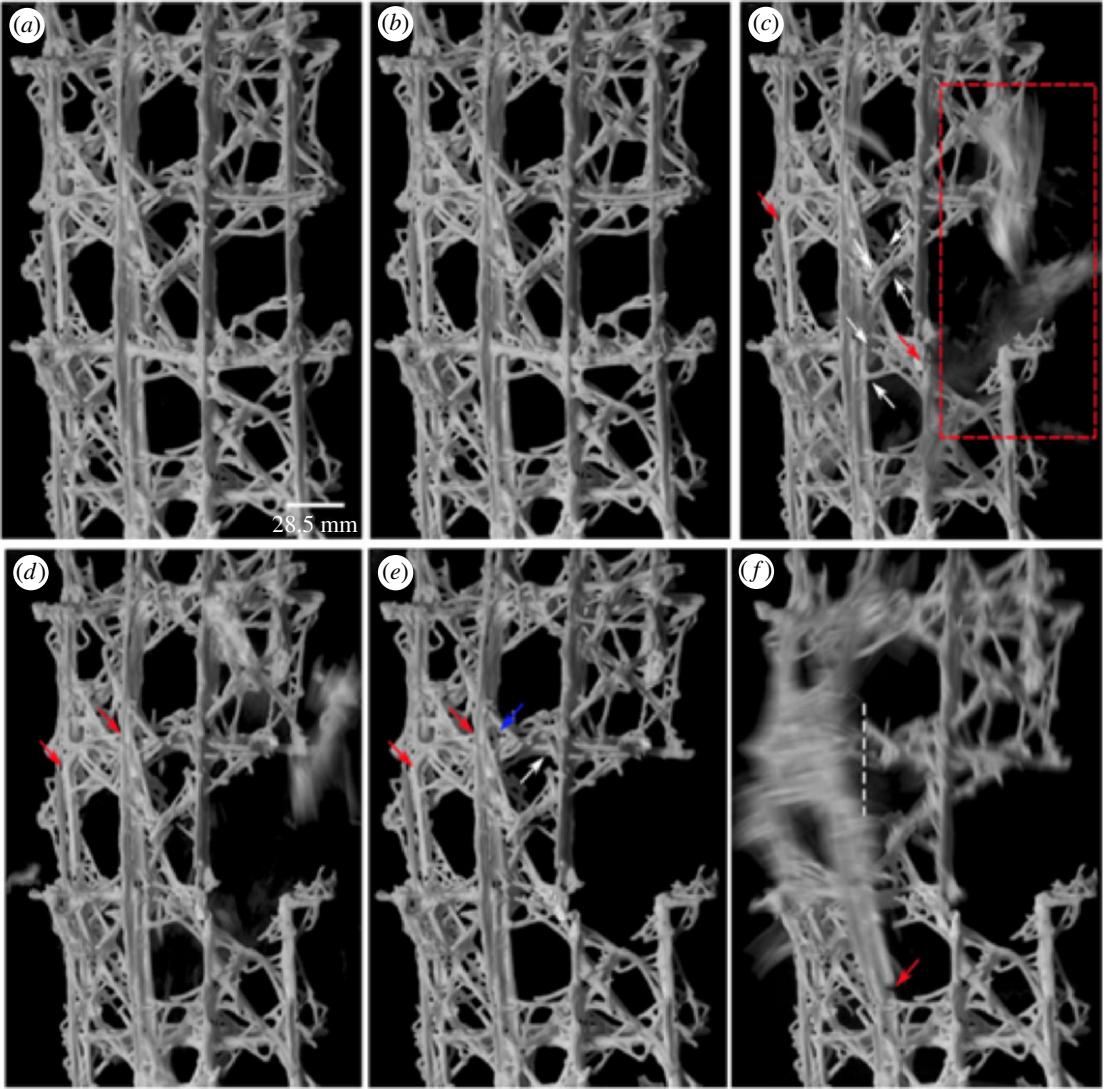
Compressive failure sequence of the 3D printed structure recorded by high-speed camera showing fracture initiation of the lattice cells and elements. (*a*) Unloaded sample; (*b*–*f*) images showing the damage development in the sample at specific points throughout the test. (Online version in colour.)

**Figure 9.**
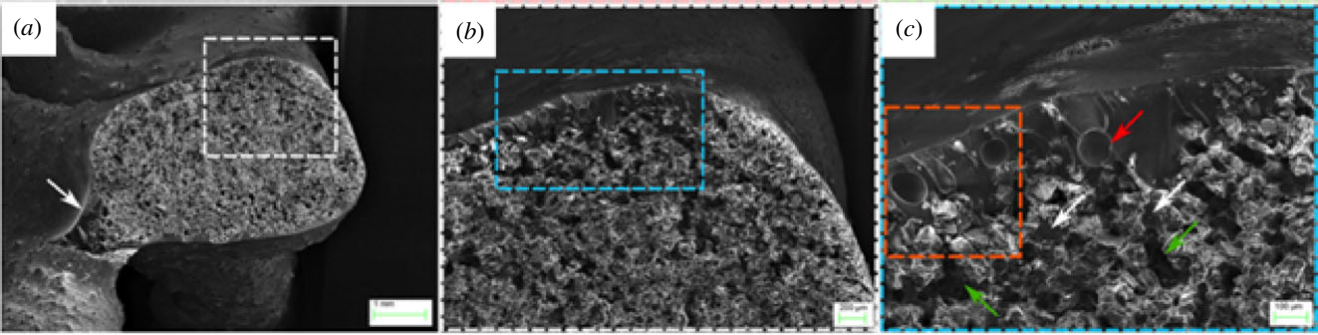
Fracture behaviour of 3D printed sample. (*a*) The presence of larger quantities of resin is clearly visible on the beam's surface (white arrow). (*b*) Magnified region of (*a*) (white dashed rectangle) demonstrating the resin infusion within inner and outer portions of the beam. (*c*) Magnified region of (*b*) showing the prevalence of intergranular fracture as a deformation mechanism. White and green arrows illustrate the fracture through the resin and the existence of voids, despite the increased quantities of resin present in the microstructure. (Online version in colour.)

The load–strain and failure sequence of the 3D printed specimen is shown in figures 7 and 8. The steep gradient of the elastic region corresponds to the stiff response of the 3D printed sample when subjected to compression loading. Such a response is observed in stretching-dominated lattices [12]. As noted in figure 7, initial failure was followed by a very sharp load drop in the outer beams (1 and 4) and subsequent load redistribution in the inner beams (2 and 3) near the yield point of the lattice framework. The sequence of photographs in figure 8 shows the failure sequence of the lattice. The first macroscopic fracture event took place at the onset of the strain-softening region in two outer beams. The sudden and explosive spalling of individual fragments from the vertical beam on the right (figure 8*c*, red dashed rectangle) can be clearly seen in figure 7 with the abrupt loss of strain targets 7–8 (figure 7*d*) and the redistribution of the load across the remaining vertical struts (targets 3–4 and 5–6 in figure 7). Simultaneously, brittle fracture in the left vertical beam also occurred (recorded as a load drop for strain target 1–2 in figure 7 and indicated as a red arrow in figure 8*c*). Additional modes of failure, i.e. crack propagation near the lattice node in the vertical beam on the left, and the extensive fracture in the diagonal reinforcing beams in the middle portion of the sample (white arrows), were also visibly notable. The continued loading of the sample resulted in additional ejection of the lattice fragments in the same region as in figure 8*c* with an increase in strain in beams 2 and 3; followed by another sharp load drop to approximately 1000 N. At 802 N, there is a slight rotation of the sample in the outward direction due to the redistribution of the load once again, resulting in further increase in strain. The fracture at the nodes between the middle and upper cell row progressed leading to final failure (figure 8*e*, red arrow). Densification of the lattice was not observed after post-yield softening. The 3D printed samples resulted in three distinct types of response to uniaxial compression: defined as predominantly brittle, predominantly quasi-brittle and soft quasi-brittle. A typical failure pattern of one of the 3D printed spicules is given in figure 9. Figure 9*a* illustrates the granular nature of the spicule architecture encapsulated with an epoxy resin infused skin. While, figure 9*b*,*c* highlights the prevalence of intergranular fracture as the primary deformation mechanism in these synthetic specimens. Fracture was initiated through the existence of voids within the 3D printed architecture, despite the addition of the infused resin present in the microstructure. All samples exhibited similar behaviour in terms of the locations of fracture initiation and progressive distributed failure.

### 4.3. Failure simulation of the 3D printed engineering analogue

The FE model was based on surface topology of the 3 × 3 Closed Cell 1 (figure 2*a*) incorporating the mechanical properties of the material used for the manufacture of the 3D printed physical samples. To enhance the prediction quality of the model, local topological transformations and automatic mesh optimization techniques were implemented to substantially reduce the number of distorted elements. This resulted in the elimination of any potential numerical instabilities occurring during the analysis.

**Figure 10.**
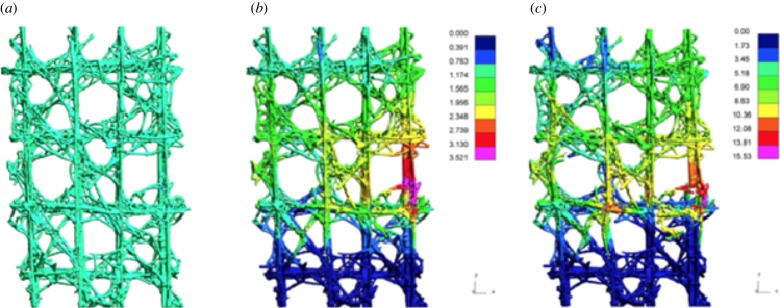
Finite-element simulation. Fringe plots of the displacement resultant at different simulation times: (*a*) unloaded, (*b*) 1.0 s and (*c*) 1.5 s. The displayed images show the front of the 3 × 3 Closed Cell model. (Online version in colour.)

The simulation results are captured in figure 10 for different simulation times, i.e. prior to loading, after 1 s and after 1.5 s. The simulation captures the initial linear response up to the first yield point, and subsequent strain hardening (hardening plasticity response) of the lattice framework until the maximum strength is attained. In the post-peak region, softening takes place until the residual strength of the material is reached [21]. It should be noted that while the KCC failure criteria have been developed specifically for the quasi-static and high strain blast response of concrete [22–24], the simulation was sufficiently robust to capture the elastic deformation mechanisms, and the quasi-brittle softening behaviour of the epoxy infused Zcorp zp150 powder. This success is despite the dimensions of the micro- and the mesoscale structural features differing significantly. A key feature arising from the FE simulations was their effectiveness at representing the observed macroscopic failure behaviour of the closed cell 3D printed samples. However, a difference of approximately 1500 N occurred between the maximum load values predicted by the FE simulations and the experimental results. The authors believe this was largely due to the employment of 4-node linear tetrahedral elements which exhibit a considerably stiffer behaviour compared to the quadratic solid tetrahedra [25]. Moreover, the applied conservative boundary conditions, and the contact modelling definitions (see the electronic supplementary material) were also accountable for the higher peak load values.

While acknowledging the limitation of capturing the precise failure load of the 3D printed architectures, the simulation results have shown that there is indeed a synergy between the biological sample (figure 5*d*) and its engineered equivalent (figure 10*c*) in terms of global compression response and the locations of damage initiation and evolution. The new scientific understanding of the architecture, connectivity and failure response developed through the simulation of the biological system can now be extracted and employed in the development of a bioinspired engineering equivalent lattice structures. In this regard, the role of structural topology and multi-layered structs and joints has been shown to be critical in the design of closed cell configurations when subjected to compression loading.

## 5. Discussion

As detailed in the hypothesis, the overarching goal of this study was to investigate the translation of a biological lattice framework (siliceous skeleton of *E. aspergillum*) to an engineering analogue. This was achieved through the application of μ-CT imaging which was used to generate both an FE model and a scaled 3D printed construct. A state-of-the art additive layer manufacturing technique employing fused ceramic particulate was used to construct global architecture. This methodology was pursued to determine if the deformation mechanisms of the biological 3 × 3 unit cell translate to its engineering scaled equivalent and whether the compressive failure behaviour of the lattice can be defined as ‘material or structure’ dominated.

The siliceous skeleton of *E. aspergillum* is characterized with an elaborate hierarchical organization, in which each hierarchy is tuned to a specific function. The multi-layered arrangement of the struts and the fusion strategies between individual struts (figure 1*d*) improve the local energy-absorbing capabilities, while the subsequent sub-structural design of irregular spicule–nodal point overlap (figure 1*b*,*c*) imparts global strength and stiffness across the whole structure.

**Figure 11.**
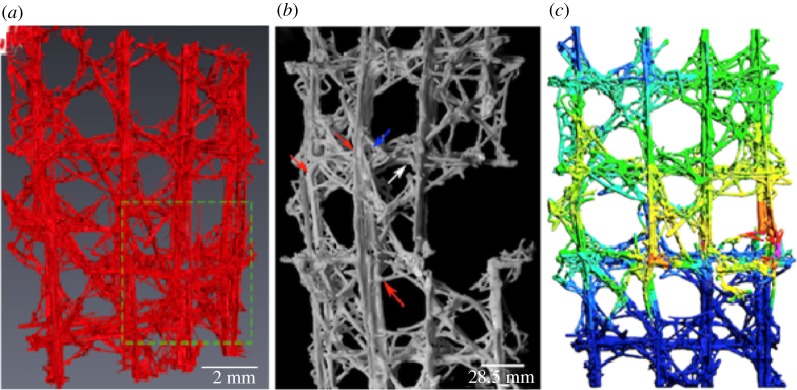
*E. aspergillum*: (*a*) X-ray μ-CT of *in situ* compression testing; (*b*) failure pattern of 3D printed engineering analogue; (*c*) finite-element simulation showing fringe plots detailing failure location of the engineering analogue. (Online version in colour.)

In comparison, the 3D printed sample is constructed with a homogeneous material (comprising bonded ceramic granular particles with an outer epoxy infused shell), with limited hierarchical organization, triggering the assumption that the mechanical response of the sample would follow conventional understanding and would follow a brittle and less damage tolerant failure response. However, the engineering analogues showed a very similar initiation and propagation failure pattern to the biological specimen when subjected to compression loading (figure 11). These experimental results suggest that the complex biological lattice design strategy can be adopted and will ‘operate’ at an engineering length scale. For this philosophy to be fully successful, improved material science understanding of the ceramic materials for AM is also required. It is through this new understanding, and the ability to manipulate and tailor the engineering materials and structure at different length scales which can ‘control’ the macroscopic deformation behaviour of the synthetic specimens (from predominantly brittle to quasi-brittle failure mode).

However, a key caveat should be acknowledged before AM is considered as the champion of bioinspired design; namely, the application of the AM process permits user selection and alignment of the printing pathways to match the primary structural loads. In this study, the printing layer orientation at the macroscale was selected to ensure the maximum performance of the printed samples under uniaxial compression. While this has maximized the uniaxial performance of the lattice framework, the off-axis performance (i.e. diagonal reinforcing struts) may have been compromised, triggering the observed quasi-brittle response. While an artefact of the engineering manufacturing process, ironically this is exactly the observed mechanism (i.e. decoupling of the fused secondary structs) which provides the damage tolerant response observed in siliceous skeleton of *E. aspergillum.* In this regard, the response has not been designed or optimized but rather an artefact of the engineering manufacturing process.

The generation of the FE simulation was very challenging. This stems from the architectural complexity and the material model to predict failure. The selection of the material model for the FE analysis was of fundamental importance to correctly capture the damage evolution, the transition from linear elastic to nonlinear elastic response and the strain-softening behaviour, exhibited by the 3D printed samples. To achieve this goal, the KCC damage model [17] was selected for the FE simulations, to capture the quasi-brittle behaviour of the closed cell 3D printed samples. KCC model is a damage plasticity model that incorporates three fixed surfaces, elastic and plastic updates, strength surface formulations and damage evolution parameters. The inherent advantage of this approach is its capability to generate fit parameters for different strength concrete material solely based on the compression strength and the density. It should be noted that despite the similarities in the quasi-brittle compression response between concrete and the resin-infused Zcorp zp150 powder, there are significant differences in their structure at micro and meso levels. These differences are directly related to the size of the aggregate and thus the deformation mechanisms responsible for the initiation and development of damage. Therefore, it was expected that the default input materials have a certain effect on the transition between the initial yield, maximum strength and residual surfaces as well as on plasticity behaviour of the closed cell FE model. Critically, the mesh density, the implemented failure criterion, the aggregate size and the contact stiffness were shown to have an effect on the plasticity and the maximum load. The difference in the response of the FE model compared to the 3D samples in terms of peak load and compressive strain can be attributed to the more conservative boundary conditions, the incorporation of linear solid tetrahedrons (which are generally stiffer compared to quadratic tetrahedrons), the default input material parameters, the failure criteria and the strain extraction techniques.

Despite the aforementioned differences, the FE simulation and the experimental results of the 3D printed samples showed good agreement in terms of the location of damage initiation and progression of failure. It was shown that the FE model efficiently represented the macroscopic failure behaviour observed in the closed cell 3D printed samples, despite the significant differences in the compression response of the latter. The simulation results showed that there is indeed overall agreement between the biological sample and its engineered equivalent, in terms of global compression response and the locations of damage initiation and evolution (figure 11). However, the rate of failure and the deformation mechanisms, controlling the accumulation of damage in each configuration, have been observed to be principally governed by the material phases and their geometrical arrangement at the smaller length scales. Realistically, until the current AM processes can actively construct a multi-material, multi-stiffness, multi-layered hierarchical architecture, rather than generating an isotropic construct with fully rigid structural linkages, the ability to mimic the tailored structural strut and joint connectivity, as observed in *E. aspergillum*, as an efficient energy-absorbing mechanism for lattice frameworks remains aspirational.

## 6. Conclusion

In the hypothesis, we set out the challenge of the current work; namely can current engineering materials in conjunction with a ‘bioinspired design philosophy’ develop the products of the future? In our efforts to answer this question, the authors have employed the latest state-of-the-art developments in μ-CT characterization, 3D AM techniques with ceramic materials and FE simulation capabilities with user derived failure criteria. A number of key conclusions have arisen from this methodology.

The structural efficiency of the lattice skeleton of hexactinellid sponge *Euplectella aspergillum* was evaluated to extract its design principles, and to translate this methodology to a commercial engineering environment. The siliceous skeleton of *E. aspergillum* is characterized with an elaborate hierarchical organization, in which each hierarchy is tuned to a specific function. While the multi-layered arrangement of the struts, consisting of hard and soft phases, and the fusion strategies improve the energy-absorbing capabilities, the subsequent levels impart strength and stiffness to the whole structure.

Notwithstanding the less elaborate structural hierarchy of the 3DP samples, comprising brittle particulate phase reinforced with another brittle phase, the experimental results clearly demonstrated that the manipulation of material and structure at different length scales altered the macroscopic deformation behaviour from predominantly brittle to quasi-brittle. The imparted enhanced damage tolerance and the subsequent ‘graceful’ failure were largely a result of the material and structural gradation of the lattice components. The observed trend strongly suggests that the compression response of the closed cell lattice arrangement was largely governed by the structure. This highlights the potential of biomimicry of the global architecture (and not biomimicry of the local material constituents) where the failure behaviour is dominated by the structure rather than a material-dominated response.

## Supplementary Material

Siliceous skeleton of E. aspergillum

## Data accessibility

Data are available from the Dryad Digital Repository at: https://doi.org/10.5061/dryad.ns7n1/1.

## References

[1] Ye L, Lu Y, Su Z, Meng G 2005 Functionalized composite structures for new generation airframes: a review. Compos. Sci. Technol. 65, 1436–1446. (10.1016/j.compscitech.2004.12.015)

[2] Gibson RF 2010 A review of recent research on mechanics of multifunctional composite materials and structures. Compos. Struct. 92, 2793–2810. (10.1016/j.compstruct.2010.05.003)

[3] Espinosa HD, Rim JE, Barthelat F, Buehler MJ 2009 Merger of structure and material in nacre and bone: perspectives on de novo biomimetic materials. Prog. Mater. Sci. 54, 1059–1100. (10.1016/j.pmatsci.2009.05.001)

[4] Dunlop JWC, Fratzl P 2010 Biological composites. Annu. Rev. Mater. Res. 40, 1–24. (10.1146/annurev-matsci-070909-104421)

[5] Dunlop JWC, Weinkamer R, Fratzl P 2011 Artful interfaces within biological materials. Mater. Today 14, 70–78. (10.1016/S1369-7021(11)70056-6)

[6] Aizenberg J, Weaver JC, Thanawala MS, Sundar VC, Morse DE, Fratzl P 2005 Skeleton of *Euplectella* sp.: structural hierarchy from the nanoscale to the macroscale. Science 309, 275–278. (10.1126/science.1112255) 16002612

[7] Weiner S, Addadi L, Wagner HD 2000 Materials design in biology. Mater. Sci. Eng. C 11, 1–8. (10.1016/S0928-4931(00)00141-7)

[8] Weaver JC et al. 2007 Hierarchical assembly of the siliceous skeletal lattice of the hexactinellid sponge *Euplectella aspergillum*. J. Struct. Biol. 158, 93–106. (10.1016/j.jsb.2006.10.027) 17175169

[9] Monn MA, Weaver JC, Zhang T, Aizenberg J, Kesari H 2015 New functional insights into the internal architecture of the laminated anchor spicules of *Euplectella aspergillum*. Proc. Natl Acad. Sci. USA 112, 4976–4981. (10.1073/pnas.1415502112) 25848003 PMC4413295

[10] Hutchinson RG, Fleck NA 2006 The structural performance of the periodic truss. J. Mech. Phys. Solids 54, 756–782. (10.1016/j.jmps.2005.10.008)

[11] Z Corporation. 2007 ZPTM60 binder: material safety data sheet. Burlington, MA: Z Corporation.

[12] UVX Extensometer Modules. 2019 See https://www.imetrum.com/documents/video-extensometer-uvx-details.pdf.

[13] Elsayed MSA, Pasini D 2010 Analysis of the elastostatic specific stiffness of 2D stretching dominated lattice materials. Mechanics of Materials 42, 709–725.

[14] Patran MSC 2012 User's guide. MSC software. Santa Ana, CA.

[15] LS-DYNA-vol.2. 2012 LS-DYNA Keyword User's Manual. Livermore Software Technology Corporation, Livermore, California, USA, version 971 edition, August 2012.

[16] Magallanes JM, Wu Y, Malvar LI, Crawford JE 2010 Recent improvements to release III of the K&C concrete model. In *11th Int. LS-DYNA Users Conference, 12–13th October*, pp 3–48. Detroit, Dynalook.com. https://www.dynalook.com/conferences/international-conf-2010.

[17] Schwer LE, Malvar LJ 2005 Simplified concrete modeling with* MAT CONCRETE DAMAGE REL3. JRI LS-Dyna User Week.

[18] Maxwell JC 1864 *Philosophical magazine* vol. 27.

[19] Pellegrino S, Calladine CR 1986 Matrix analysis of statically and kinematically indeterminate frameworks. Int. J. Solids Struct. 22, 409–428. (10.1016/0020-7683(86)90014-4)

[20] Elsayed MSA, Pasini D 2010 Analysis of the elastostatic specific stiffness of 2D stretching-dominated lattice materials. Mech. Mater. 42, 709–725. (10.1016/j.mechmat.2010.05.003)

[21] Brannon Rm, Leelavanichkul S 2009 Survey of four damage models for concrete. Sandia National Laboratories, US Department of Energy, Oakridge, TN 37831, USA. http://www.mech.utah.edu/∼brannon/pubs/7-2009BrannonLeelavanichkulSurveyConcrete.pdf.

[22] Noble C, Kokko E, Darnell I, Dunn T, Hagler L, Leininger L 2005 Concrete model descriptions and summary of benchmark studies for blast effects simulations. Technical report. Livermore, CA: Lawrence Livermore National Laboratory (LLNL).

[23] Crawford JE, Malvar LJ, Wesevich JW, Valancius J, Reynolds AD 1997 Retrofit of reinforced concrete structures to resist blast effects. ACI Struct. J. 94, 29 (10.14359/488)

[24] Crawford JE, Wu Y, Choi H-J, Magallanes JM, Lan S 2012 Use and validation of the release iii concrete material model in ls-dyna. Technical report TR-11-36.5. Glendale, CA: Karagozian & Case.

[25] Cook RD 1994 Finite element modeling for stress analysis. New York: John Wiley & Sons.

