# Supplementary material for: The structural efficiency of the sea sponge Euplectella aspergillum skeleton: bio-inspiration for 3D printed architectures
Source: J R Soc Interface. 2019 May 8;16(154):20180965. doi: 10.1098/rsif.2018.0965 (PMC6544886; doi:10.1098/rsif.2018.0965)

**The structural efficiency of the sea sponge *Euplectella aspergillum* skeleton: bio-inspiration for 3D printed architectures.**

*K. Robson Brown^1, 2^, D. Bacheva^3^, R.S. Trask^4^

^1^ CT Imaging Laboratory, 43 Woodland Road, University of Bristol, Bristol, BS8 1UU, UK.

^2^ Department of Mechanical Engineering, University of Bristol, University Walk, Bristol, BS8 1TR, UK

^3^ Hieta Technologies, Bristol and Bath Science Park, Bristol BS16 7FR, UK.

^4^ Department of Aerospace Engineering, Advanced Composites Centre for Innovation and Science,

University of Bristol, University Walk, Bristol, BS8 1TR, UK

**Supplementary Information**

**Siliceous skeleton of E. aspergillum - sample extraction and preparation**

Samples of the closed cell configuration, were extracted from the lower portions of several E. aspergillum specimens, were manually extracted using a sharp scalpel. To remove any remaining debris, the samples were washed in distilled water, cleaned for 30 sec in an ultrasonic cleaner and dried at room temperature for 24 hours. One of the advantages of μCT is that it requires only minimal sample preparation. The cleaning of the samples was conducted to perform a post-in situ SEM analysis on the fractured samples. To secure the upright position of the samples and to ensure flat and parallel surfaces for testing, the upper and lower ends were cast in epoxy resin EPON 828 with diethylenetriamine (DETA) as curing agent using a silicon mould. The casting of the samples was performed at room temperature, and within two hours of curing to prevent an upward transfer of resin through the spicules.

**Siliceous skeleton of E. aspergillum - micro-CT scanning.**

SkyScan 1172, a high resolution μCT scanner (SkyScan, Belgium) was used in all experiments. Scanning all samples at resolution 4.91 μm isotropic voxel prior to loading and after each loading cycle, X-ray radiation with a peak energy of 68 kV, current of 153 mA, an angular step of 0.280 deg over 180 rotation and number of projections equal to 730. In addition, frame averaging was applied to increase the signal-to-noise ratio and thus to improve the image quality. In the former, each projection is imaged several times. The average of all projections is then used for image reconstruction. The average acquisition time of the scans after was 1h 25 min.

**Siliceous skeleton of E. aspergillum - in-situ testing micro-CT scanning.**

Each sample was mounted in the specimen chamber of SkyScan Material Testing Stage (MTS-50N) within the SkyScan 1172. The testing stage consist of the following components. The enclosed bottom part (1) houses a microprocessor controller, a port which connects it to specimen chamber of the μCT scanner, a stepper motor drive with a gear box and solid-state force sensor. The output gearhead (2) imparts a rotation to two coaxial central shafts, coming from the top of the gearhead and positioned one above each other. The shafts rotate with equal speed but in opposite direction, hence permitting the application of force at the top and the bottom of the samples. During compression, the bottom object holder (4), connected to the upper central shaft, moves upwards. The clear specimen chamber (5), together with the supporting metal ring (3) and the top object holder (6) moves downwards during compression, creating a uniform compression loading in the central part of the sample [SkyScan, 2007]. The individual samples were loaded in compression with a rate of 2 μm/s in several incremental steps. All scans were conducted using the parameters detailed in the previous section.

**Figure S1**: Bruker SkyScan Material testing stage (MTS-50N). (A) Components of the stage:

(1) Bottom part, (2) Gearhead, (3) Metal supporting disk, (4) Bottom object holder,

(5) Clear specimen chamber, (6) Top object holder. (B) and (C) Sequence of mounting the samples in the chamber of the in situ testing stage.

**Siliceous skeleton of E. aspergillum - scanning electron microscopy**

Individual samples were mounted on aluminium stubs using conductive carbon tabs. Subsequently, the samples were sputter coated with silver and examined with Zeiss EVO MA 25 (ZEISS, Oberkochen, Germany) scanning electron microscope in high vacuum mode.

**3D printed specimens parameters**

Dimensions [mm] 194.77 x 294.11 x 64.64

Layer thickness [mm] 0.085

Number of layers 760

Print time 4 h 29 min

**Finite Element Input Parameters**


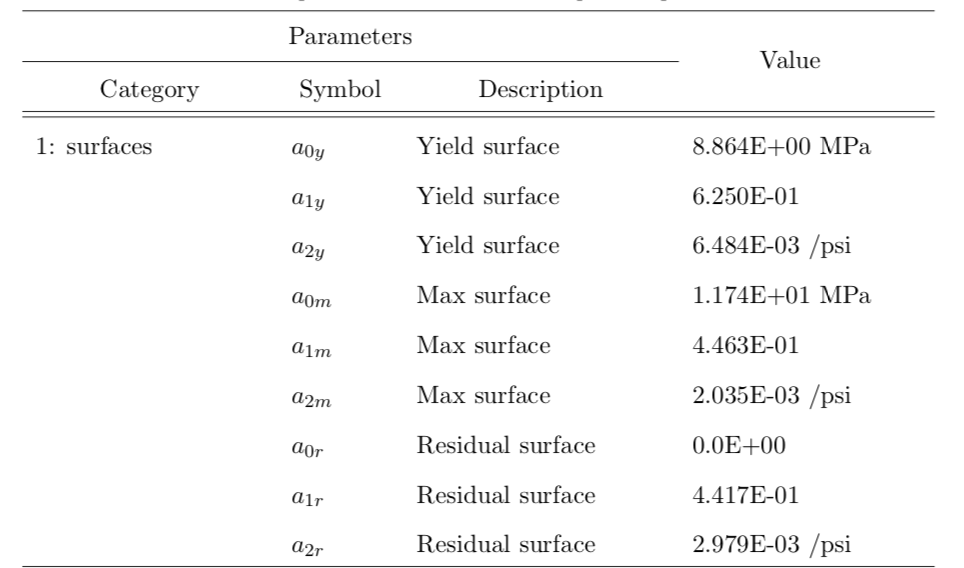

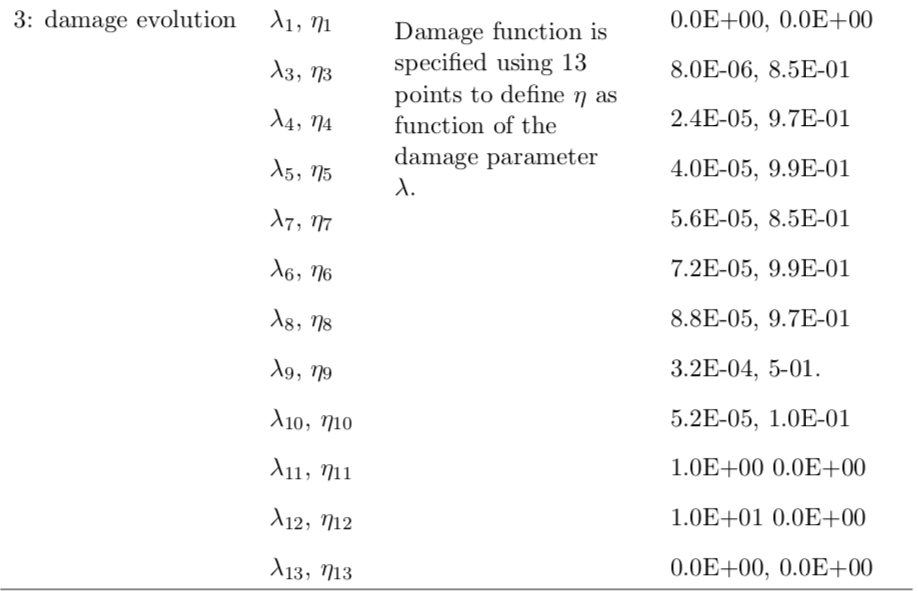

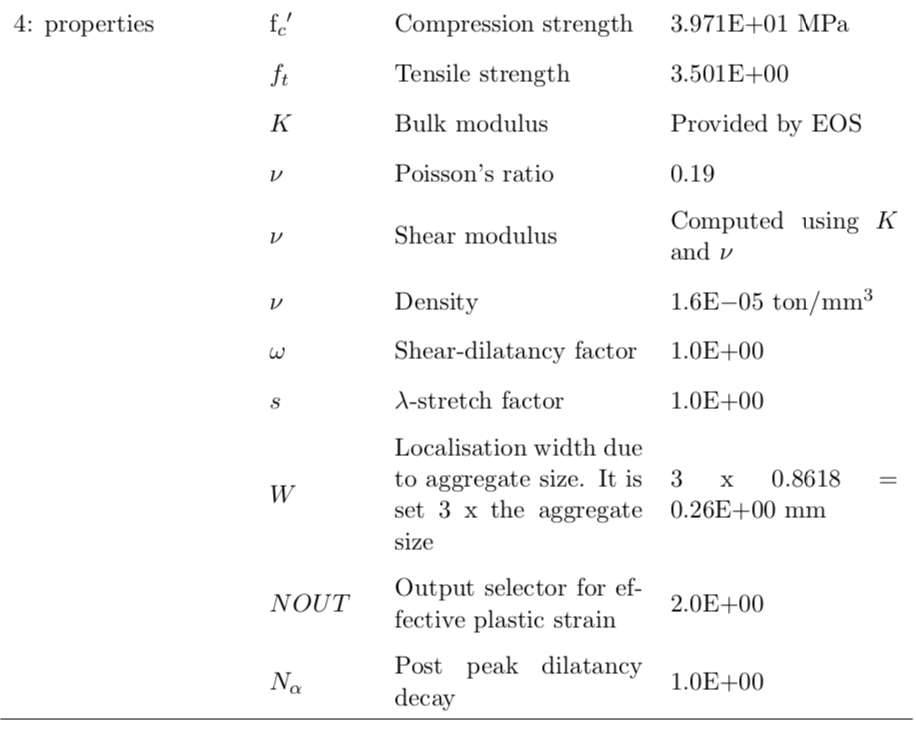

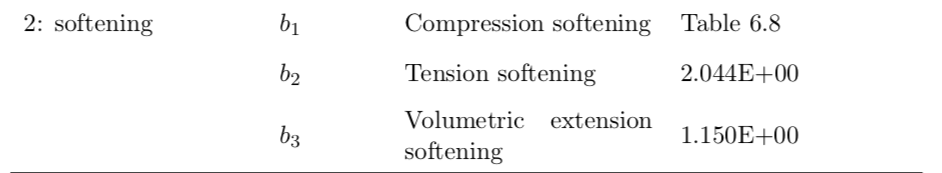


0.810E+00

**Application of global translational and rotational constraints in FEA**

The boundary conditions concern the top plate and the 3 x 3 Closed Cell model. The top plate was modelled with five constraints. There were no translations along the model global x and y axes and no rotations around the three global axes. Therefore, only a downward displacement along the z was permitted with a constant velocity of 2.5 mm/sec.


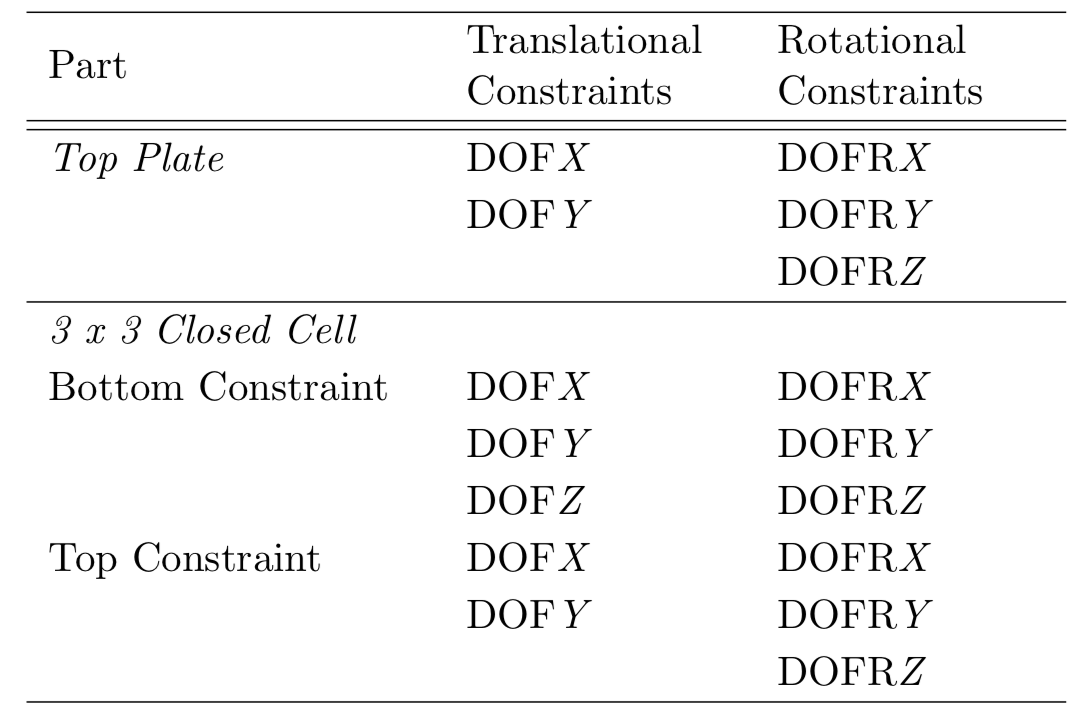

Supplement: Siliceous skeleton of E. aspergillum [file rsif20180965supp1.docx]
